# HIV Self-Testing to Promote Serostatus Disclosure Among Men Who Have Sex With Men in China: Protocol for a Stepped Wedge Randomized Controlled Trial

**DOI:** 10.2196/17788

**Published:** 2020-07-09

**Authors:** Tianyi Lu, Hang Li, Xiang Mao, Erlei Peng, Yangyang Gao, Zhenxing Chu, Jing Zhang, Willa Dong, Yongjun Jiang, Junjie Xu

**Affiliations:** 1 NHC Key Laboratory of AIDS Immunology Department of Laboratory Medicine First Affiliated Hospital of China Medical University Shenyang China; 2 Department of Health Behavior Gillings School of Global Public Health University of North Carolina at Chapel Hill Chapel Hill, NC United States

**Keywords:** HIV, HIV self-testing (HIVST), HIV serostatus disclosure, men who have sex with men (MSM), stepped wedge randomized controlled trial, China

## Abstract

**Background:**

Disclosure of HIV serostatus is important for the prevention of HIV infection among men who have sex with men (MSM). However, knowledge of sexual partners’ HIV status among MSM in China is low. As a complement to HIV testing services, HIV self-testing (HIVST) has considerable potential to promote serostatus disclosure.

**Objective:**

The primary objective of our trial is to evaluate the effect of HIVST on improving serostatus disclosure to sexual partners. We hypothesize that MSM in an intervention condition will have a higher awareness of the HIV status of their sexual partners compared with MSM in the control condition. The secondary aims are to evaluate (i) changes in sexual behaviors after disclosure of HIV status by sexual partners, (ii) promotion of the frequency of HIV and syphilis testing on participants and their sexual partners, and (iii) factors that restrict the disclosure of HIV infection to sexual partners. We hypothesize that MSM in the intervention condition will exhibit safer sexual decision making and a higher rate of HIV testing uptake compared with MSM in the control condition.

**Methods:**

A stepped wedge randomized controlled trial will be conducted throughout China. Study recruitment of 800 MSM will be promoted through advertisements released on WeChat public accounts. Individuals who are born biologically male, aged ≥18 years, HIV negative, and who have not undergone HIV testing in the past 3 months will be recruited. Eligible men will be randomly divided (1:1:1:1) into four groups and randomized. The group cluster will initiate the intervention so that participants will be provided with 2-4 free finger prick–based HIVST kits until trial completion. The intervention period for participants in each of the four groups will be initiated at 3-month intervals. Men in both groups will be required to complete a baseline and four follow-up surveys every 3 months. The primary intervention outcome will evaluate the effect of the distribution of HIVST kits on improvement in the disclosure of sexual partners’ HIV status. The secondary outcomes will be changes in sexual behaviors after disclosure of HIV status from sexual partners, the promotion of the frequency of HIVST on participants and their sexual partners, and the factors that restrict disclosure of HIV status to sexual partners.

**Results:**

Subject recruitment began in August 2018. The first round of follow-up surveys post intervention is complete, with three rounds remaining to be done. Data analysis was scheduled for April 2020 and the results will be disseminated through conferences and peer-reviewed publications.

**Conclusions:**

Few studies have evaluated interventions to increase knowledge of sexual partners’ HIV status among MSM. Our trial will provide information on the link between HIVST and HIV serostatus disclosure. The findings of this trial will facilitate the implementation of HIVST services to help control the spread of HIV among MSM in China.

**Trial Registration:**

Chinese Clinical Trial Registry ChiCTR1800019453; http://www.chictr.org.cn/showproj.aspx?proj=30158

**International Registered Report Identifier (IRRID):**

DERR1-10.2196/17788

## Introduction

### Background

The HIV epidemic is a significant public health problem worldwide, especially among men who have sex with men (MSM) [1,2]. Of all new HIV infections worldwide in 2017, MSM accounted for 18% but reached 29% in the Asia-Pacific region [3]. National surveillance data have indicated that HIV prevalence among MSM in China increased from 0.9% in 2003 to 8% in 2015 [4,5]. Despite considerable efforts, HIV-positive MSM comprise an increasing proportion of newly infected individuals infected through regular or casual sexual partners [6]. Thus, HIV serostatus disclosure from sexual partners is essential for the prevention and control of HIV infection among MSM [7].

There are several benefits of HIV serostatus disclosure between sexual partners. It facilitates serosorting, promotes safer sexual behavior, and reduces the risk of HIV acquisition [8-10]. For example, women in Kenya are more likely to use condoms after receiving the HIV-positive results of male partners [11]. However, the practice of HIV serostatus disclosure remains uncommon among MSM in low- and middle-income countries (LMICs) [12,13]. A recent study among MSM in China found that the knowledge of sexual partners’ HIV status for most “regular” and “casual” male partners was only 20.6% and 17.8%, respectively [14]. This low prevalence may be due to fear of violence [15], concerns about upsetting family numbers and rejection [16,17], or lack of social support [18]. Low uptake of HIV testing is another critical concern with the disclosure of HIV status [19]. However, a meta-analysis in China showed that only 38% of MSM had taken an HIV test in the previous 12 months and that 53% of MSM had never undertaken an HIV test in their lifetime [20].

HIV self-testing (HIVST) is a complement to conventional facility-based HIV testing services and has been recommended by the World Health Organization (WHO) since 2015 [21]. Individuals who use HIVST can collect their blood or saliva privately, conduct a test, and read the results themselves. With the characteristics of privacy, convenience, and confidentiality, HIVST is attractive to MSM [22-24]. Studies have indicated that HIVST has high acceptability among MSM, and a considerable proportion of MSM have expressed great interest and are willing to use it [25-27]. Two randomized controlled trials (RCTs) conducted in developed countries demonstrated that HIVST increases the frequency of HIV testing among MSM [28,29].

Many studies have primarily focused on the process [30], benefits and barriers [16], and factors associated with HIV serostatus disclosure [9]. One prospective cohort study and an RCT in Kenya indicated that HIVST kits distributed by sexually active females could promote HIV status disclosure from their heterosexual partners, and subsequently safer sexual decisionmaking [11,31]. However, few studies have explored the intervention effect of HIVST to promote HIV status disclosure among MSM. Further studies are urgently needed to fill the current gap and evaluate the effect of HIVST intervention on improvement of HIV serostatus disclosure among MSM in China.

### Objective

The primary objective of our trial is to evaluate the effect of HIVST on improving HIV serostatus disclosure between sexual partners. The hypothesis is that MSM receiving free HIVST test kits and counseling will have a higher awareness of the HIV status of their sexual partners compared with MSM in the control condition. The secondary aims are to determine the: (i) changes in sexual behaviors after disclosure of HIV status from sexual partners; (ii) promotion of the frequency of HIV and syphilis testing on participants and their sexual partners; (iii) factors that restrict the disclosure of HIV infection to sexual partners. The hypotheses are that MSM in the intervention condition will exhibit safer sexual decisionmaking and a higher rate of HIV testing compared with MSM in the control condition.

## Methods

### Trial Design

Given the significantly higher risk of acquiring HIV among MSM than the general heterosexual population, an RCT is not appropriate on ethical grounds. Therefore, this study was designed as a stepped wedge RCT. This design will ensure that all participants have an equal chance to initiate the intervention, though each group will begin the intervention at different times. Also, a pragmatic stepped wedge RCT design will allow the evaluation of the intervention in different groups rather than in a single group.

### Study Setting and Recruitment

An online cohort will be established. All information, including surveys and HIVST results, will be collected through Gold Data, an online platform dedicated to data collection [32]. A few personal WeChat (Tencent) public accounts that are active in the MSM social circle will release an advertisement to invite MSM to participate in the trial. The advertisement will explain the study objectives, content, and procedures. Restrictions on the use of Internet Protocol addresses will ensure that all participants can complete the survey only once. A 20 RMB (2.84 USD) incentive will be delivered to each participant for the first baseline, and 20 RMB (2.84 USD) for each follow up. Participants who complete all five surveys (baseline and four follow-ups) will earn an extra 200 RMB (28.39 USD) to compensate for their time.

A baseline survey will be used to assess eligibility. Eligible men will be randomly divided into four groups at a ratio of 1:1:1:1. Intervention for the four groups will be randomized and initiated at 3-month intervals. Participants who reach the intervention period will be provided with 2-4 free HIVST kits. The trial will last for 1 year. All individuals who enroll in the trial will be required to complete four follow-up surveys every 3 months. The trial will not be blinded because researchers will be aware of the assignment and sequence with which intervention materials are delivered to each participant. A flowchart describing the trial design is presented in Table 1.

**Table 1:** Study schedule.

Timepoint		Enrollment		Allocation		Post allocation						
**Enrollment**		Aug 2018-Feb 2019		Aug 2018-Feb 2019		T1^a^		T2^b^	T3^c^		T4^d^	
	Online recruitment		X									
	Eligibility screen		X									
	Informed consent		X									
	Allocation				X							
**Intervention initiated**												
	Group A						I^e^	I		I		I
	Group B						C^f^	I		I		I
	Group C						C	C		I		I
	Group D						C	C		C		I
**Assessments**												
	Online surveys						X	X		X		X

^a^T1: 1-3 months after enrollment.

^b^T2: 3-6 months after enrollment.

^c^T3: 6-9 months after enrollment.

^d^T4: 9-12 months after enrollment.

^e^I: Intervention condition.

^f^C: Control condition.

### Inclusion Criteria

Men who provide written informed consent and meet all of the following criteria will be regarded as eligible and invited to participate: born biologically male; age ≥18 years; HIV-negative; had oral or anal sex with men in the previous 6 months; no HIV testing in the past 3 months; willing to provide their telephone number and address (for follow-up and delivery of HIVST kits); willing to accept HIVST kits; willing to share truthful information regarding sexual behavior and HIV testing; willing to accept consultation before and after HIVST and links to HIV care.

### Exclusion Criteria

MSM will be excluded if they are participating in other research projects, unable to speak or read the Chinese language, unable to complete the online questionnaire using a mobile telephone, or cannot provide written informed consent.

### Randomization and Allocation

The Mersenne Twister pseudo-random number generator will generate 200 groups of 1-4 random number tables using SAS (SAS Institute) to determine each participant’s group assignment. Then, researchers will randomly select four balls marked 1, 2, 3, and 4 from a closed container to determine the intervention sequence (A-B-C-D). Interventions will be initiated at 3-month intervals.

### Intervention Implementation

When MSM participants reach the intervention period, researchers will send a Quick Response (QR) barcode to each participant to request delivery of 2-4 HIVST kits. The survey is nationwide, and participants will be asked to provide their telephone number and address for delivery of the requested kits.

The HIVST kits will be sent 1-2 days after request and can be used for HIV testing by the participants themselves and their sexual partners. To confirm that participants have used their HIVST kits and help researchers provide consultations, photographs of all HIVST results must be uploaded and submitted through the online survey platform within 20 min after testing. If participants have shared the HIVST kits with their sexual partners, the latter will also be asked to upload a photograph of their HIVST results and complete a questionnaire. The telephone number of participants who share the HIVST kits will also be required for indemnification matching. Participants will be permitted to apply for repeat delivery of 2-4 HIVST kits if previously delivered kits have been used, with no restrictions on the number of HIVST kits permitted during the research period. For participants’ convenience and privacy, the HIVST kits will be delivered by express mail in discreet packaging without visible text mentioning HIVST. Participants will not have to pay for HIVST kits or postage.

### HIVST Kit Components

**Figure 1:**
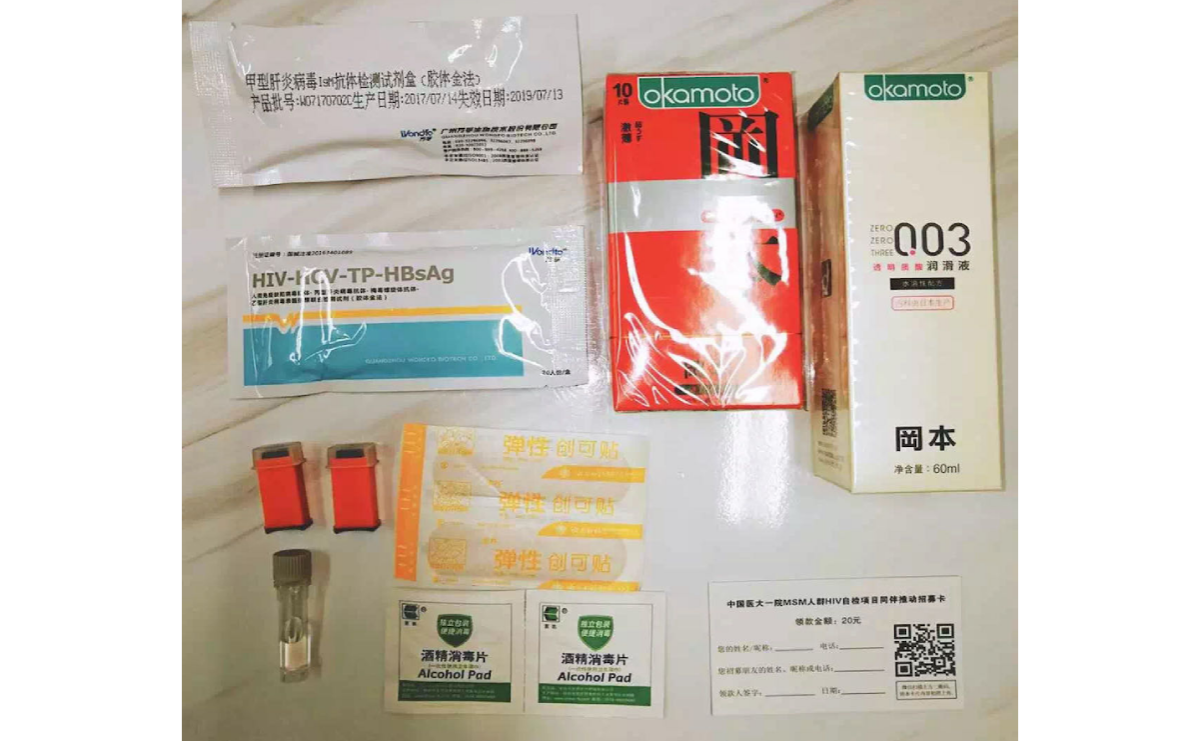
HIVST kits used in the study.

Finger prick HIVST kits (Wondfo) approved by the Food and Drug Administration of China will be used. Antibodies against HIV 1/2 will be detected 6 weeks post infection using blood specimens [33]. In addition to HIV, the assay also contains reagents to test for antibodies against syphilis, the hepatitis C virus, and surface antigens of the hepatitis B virus, which are prevalent among MSM in China (Figure 1). Consumables needed for testing (alcohol, cotton, disposable blood needles, EDTA capillary tubes, and bandages) will be provided in each kit. Video and written instructions will provide detailed procedures for testing and an explanation of possible results. Participants can contact the study administrators through a 24-h telephone number or WeChat account if they encounter problems or difficulties during testing.

### Consultation and Referral Services

Researchers will provide consultation via WeChat, the platform used to enroll in the study, before and after testing. Trained researchers will provide pretest consultation for all participants, informing them of the purpose and significance of HIV testing at the time participants make their test kit request. In addition to sending HIVST kits, test kit instructions (written and video) will be distributed. For those participants who cannot use the self-testing kits after reading the instructions, WeChat and telephone support will be provided. Posttest counseling will be provided within 24 hours after the test results are uploaded. Test results uploaded by participants and their sexual partners will be reviewed daily. Individuals whose test results are “positive” or “indeterminate” will be contacted immediately by telephone or WeChat. Posttest consultation for those who upload HIV-negative results will consist of a reminder of the meaning of a negative test result and how to prevent HIV infection. Those who have positive self-test results will receive HIV posttest consultation services, including interpretation of the test results and referral to services for clinical confirmation testing and antiviral treatment. Materials for dried-blood spot (DBS) testing will be recommended and freely provided for further testing, including western blotting and nucleic-acid detection, at the Key Laboratory of AIDS Immunology of Liaoning Province (First Affiliated Hospital of China Medical University, Shenyang, China) [34]. HIV laboratory test result confirmation will be provided to participants, and counseling and referral will be suggested.

### Contamination Control

The initial screen will ask whether the applying participant’s partners or friends have participated in this HIVST study. To reduce the potential for contamination between groups, participants who indicate their partners or friends have participated in the study will be deemed ineligible to participate.

### Study Measures and Outcomes

Baseline and follow-up surveys will be completed via mobile telephone and collected through Gold Data, the most popular online data collection system platform in China. Study administrators will establish an online survey form and generate a QR code link to the survey questionnaire. Study subjects will scan the QR code provided and complete the survey, indicating their willingness to participate in the project and the conditions for participation. A link to the questionnaire is also included in the HIVST kit instruction manual mailed to the respondent. After completing the HIVST, participants scan the QR code and provide their identification numbers, social background, and behavioral information, and uploaded photos of self-test results.

Data privacy is assured by contractual agreement with Gold Data, as well as Gold Data’s encryption systems. Participants are asked to use pseudonyms rather than their real names. A secondary identifier is provided with each participant’s questionnaire. Study administrators also used physical controls, such as locked storage for study data, to ensure participants’ privacy and data security.

A baseline questionnaire will be used to screen candidates for inclusion eligibility. Eligible participants will be required to complete the remaining questions in the surveys. Data collection will include social background (age, level of education, monthly income, occupation, marital status, and sexual orientation), high-risk sexual behaviors, and experience of HIV testing. High-risk sexual behaviors, including the number and type of sexual partners, number of condom-less oral or anal sex encounters, and knowledge of sexual partners’ HIV status during the past 3 months, will be documented. HIV testing history, including the number of self-reported HIV tests, experience with HIVST, and variables, will also be recorded.

Participants will be asked to complete follow-up surveys within one week after being notified via WeChat message. Follow-up surveys will be performed at 3, 6, 9, and 12 months after enrollment. The distribution of HIVST kits, sexual partners’ willingness to use the kits, and the rate of HIV status disclosure by sexual partners will be collected. In addition to high-risk sexual behaviors and HIV testing experience in the baseline survey, sexual decision making will also be measured to evaluate the effect of HIV status disclosure. Questions regarding sexual decisionmaking will focus mainly on whether to have sex and condom use (never/sometimes/usually/always using) for oral or anal sex after knowing the HIV status of sexual partners (negative/positive/unknown). Participants who report that they are unwilling to disclose their HIV status to sexual partners will be asked questions about sexual orientation, HIV status, type of sexual partners, HIV testing-related violence, and HIV testing history.

Reminder messages will be sent via WeChat for 3 weeks to ensure the completion of follow-up surveys. Participants will be considered lost to follow-up if they do not complete their survey within 1 month.

### Sample Size

To calculate sample size, we used the calculation tool for the stepped wedge RCT design developed by Hussey et al [35]. Tang and colleagues found that in China, the proportions of MSM being told of their partners’ HIV status were 20.6% and 17.8% for most regular and casual male partners, respectively [14]. Assuming that the effect of the intervention period was superior to that of the control period, an overall growth of 10%, four intervention periods, a coefficient of variation of 0.40 (usually between 0.15 and 0.40), a two-sided α of 0.05, and 90% power, the total sample sizes of 620 and 560 for the most recent regular and casual male partners were significant. Given a loss to follow-up of 20%, we made a conservative estimate that 200 men in each group would be sufficient to detect differences between the intervention and control conditions.

### Data Management

Survey data and photographs of testing results will be collected through an online survey platform. Only researchers will have an account and password, so the safety and privacy of participants are ensured. Data will be downloaded directly and saved in an identifiable format on a computer. When the data are transmitted, some identifiable variables, such as name, telephone number, and address, will be encrypted for privacy protection. A specific telephone will be used for daily contact with participants during the trial and will be locked in a document cabinet after work every day.

### Analysis Plan

Survey data will be imported into SAS for data management and analysis. The primary outcome will be the self-reported disclosure of HIV status by sexual partners during the past 3 months. Generalized linear mixed models will be used to compare the differences between intervention and control conditions for primary outcome analysis. Intervention status and time will be treated as fixed effects, whereas group clusters and individuals will be estimated as random effects. The effects of the intervention will be reported with 95% confidence intervals and *P* values. *P*<.05 will be considered significant.

Similar methods will be used to measure secondary outcomes, including the frequency of HIV testing uptake, frequency of syphilis testing, number of sexual partners, and unprotected sex. In addition, logistic regression analysis will be used to explore the relationship between the HIVST results of sexual partners and condom use during sexual intercourse. Factors such as sexual orientation, HIV status, type of sexual partners, HIV testing-related violence, history of HIV testing, and others will be measured to examine their association with disclosure of HIV status to sexual partners.

### Patient and Public Involvement

We invited a community-based organization worker from the Shenyang Sunny organization and two MSM to participate in revising the structure and content of our recruitment advertisement, guaranteeing it would be attractive and relevant to the MSM community. These individuals were not involved in conducting the study. As the intervention will be initiated at different times, all participants have the right to seek other HIV testing services during the study, regardless of the group to which they have been assigned. The results will be disseminated to the public and study population after study completion.

### Ethics and Dissemination

The research program and procedures have been approved by the ethics committee of the First Affiliated Hospital of China Medical University (2018-174-2). The study was registered with the Chinese Clinical Trial Registry (trial ID: ChiCTR1800019453) on November 12, 2018. Informed consent forms will be provided to each participant before the questionnaire and distribution of HIVST kits. Participants will be able to withdraw from the study at any time. We will use a mobile telephone to contact respondents. The findings of the study will be made available to local and national government agencies in China and disseminated through academic publications and international conferences.

The study was retrospectively registered under ID ChiCTR1800019453 on Chinese Clinical Trial Registry because the authors were unaware of the definition of a clinical trial per the International Committee of Medical Journal Editors.

## Results

At the time of publication, recruitment and intervention are complete for all participants. Data for the baseline and initial investigation have been added to Chinese Clinical Trial Registry.

## Discussion

HIV serostatus disclosure has important implications for the prevention and control of HIV epidemics. Studies have suggested that HIV testing (especially for couples) is critical for disclosure of HIV status [36]. The situation is similar in China. A recent observational study of eight cities showed that HIV testing and HIVST were positively associated with HIV status disclosure for regular and casual male partners [14]. Studies have demonstrated that HIVST can promote the disclosure of HIV status among heterosexual couples, but no scholars have evaluated the impact among MSM. A nationwide, large-scale, stepped wedge RCT is necessary to evaluate the ability of HIVST to facilitate HIV status disclosure among MSM in China. If successful, evidence derived from our trial could be used to expand the implementation of HIVST services. The trial outcome will also provide policy and intervention strategies for this key population in government and community organizations.

Our trial will also explore the impact of HIVST intervention on HIV testing uptake among participants and their sexual partners. Since 2012, the WHO has recommended MSM to undergo HIV testing with their partners [37]. Our trial will provide multiple HIVST kits to each participant and permit them to share those kits with their sexual partners. This form of secondary distribution may aid in our understanding of how to improve the level of HIV testing uptake in MSM.

Our trial has four main limitations. First, a 12-month research period is relatively long, and to maintain compliance from participants (especially during follow-up) will be challenging. To compensate for this anticipated loss, we will enlarge the sample size by 20%. Second, because the survey interval is 3 months, recall bias may occur during information collection. To compensate, researchers will assess whether some questionnaire variables are consistent with subsequent feedback from participants. Third, migration of participants may occur during the long study period. To circumvent this problem, we will remind participants to contact us and update their mobile telephone number and residence address. Other information will be used to match identification if necessary. Finally, participants will be recruited through the internet; MSM who lack access to the internet will not be recruited, which may limit the generalizability of study results.

## Appendix

Multimedia Appendix 1 CONSORT checklist.

## Abbreviations

HIVST: HIV self-testing
LMIC: lower- and middle-income countries
MSM: men who have sex with men
QR: quick response (barcode)
RCT: randomized controlled trial
WHO: World Health Organization
